# Mortality and transitions-of-care after COVID-19 hospitalization among US Medicare patients: a retrospective claims analysis

**DOI:** 10.1186/s12877-026-07700-7

**Published:** 2026-05-28

**Authors:** Alon Yehoshua, Rachel M. Black, Anan Zhou, Michelle A. Silver, Yu Han, Santiago MC Lopez, Manuela Di Fusco, Benjamin Yarnoff, Rajeev M. Nepal, Maria M. Fernandez, Mohammad Ashraf Chaudhary, Tara Ahi

**Affiliations:** 1 Pfizer Inc, New York, NY USA; 2 AESARA Inc, Chapel Hill, NC USA; 3 Genesis Research Group, Hoboken, NJ USA; 4 Evidera Inc, Bethesda, MD USA

**Keywords:** COVID-19, Older adults, Post-hospitalization, Mortality, Transitions-of-care

## Abstract

**Background:**

The patient burden from COVID-19 extends beyond acute hospitalization, especially for older adults. The objective of this study was to describe post-discharge care settings and mortality rates after a COVID-19 hospitalization among adults aged ≥ 65 years in the United States.

**Methods:**

This retrospective observational study used the Medicare fee-for-service dataset. We identified Medicare patients hospitalized with COVID-19 from September 2023–February 2024. The date of discharge was the index date. Patients were followed until death, end of enrollment, or six months post-index. Pre- and post- hospitalization care settings, all-cause mortality, and readmission rates were analyzed. Patients were stratified by COVID-19 severity (general ward, intensive care unit [ICU], invasive mechanical ventilation [IMV]) and age (65–74, ≥ 75 years old).

**Results:**

A total of 67,358 patients were included; most were female (55.6%), white (84.9%), and on average 80.8 years (standard deviation: 8.1). The majority (96.4%) had ≥ 1 high-risk condition as defined by the Centers for Disease Control and Prevention (CDC). The median (interquartile range) length of stay was 5.0 (3.0–7.0) days. During index hospitalization, 4.4% of patients were admitted to the ICU and 5.1% required IMV. Post-discharge, 50.5% of patients who resided at home pre-hospitalization (self-care or under care) required increased care. Less than half (47.8%) of patients who were home (self-care) pre-hospitalization returned home (self-care) upon discharge.

A total of 11,658 (17.4%) patients died within 6-months of hospital discharge. Mortality rates increased for patients requiring higher levels of care: 7.1% of patients discharged home (self-care), 13.0% of patients discharged home (under care), and 31.8% discharged to any healthcare facility died within six months. Mortality was higher in those with more severe COVID-19 and those aged ≥ 75 years. The COVID-19-related readmission rate was 4.5% within six months of discharge, and 3.2% occurred within 30 days.

**Conclusion:**

The proportion of older adults who lost independence and required care (under care at home or at a healthcare facility) more than doubled after COVID-19 hospitalization, making the post-discharge period a particularly vulnerable time for patients, who are at risk for death and hospital readmission.

**Supplementary Information:**

The online version contains supplementary material available at 10.1186/s12877-026-07700-7.

## Background

COVID-19 continues to have substantial morbidity and mortality rates, particularly in the older population [1]. In the 2023–2024 COVID-19 season (October 2023-September 2024), the overall cumulative rate of COVID-19-associated hospitalization in the United States (US) was 821.1 per 100,000 adults aged ≥ 65 years [2]. In the same age group, estimated cumulative mortality count from September 2023-August 2024 was 36,357; [3] prior studies have reported increased mortality rates due to COVID-19 in older adults compared to the pre-pandemic period [4–6]. In 2022, it was found that COVID-19 deaths occur primarily during hospitalization (59%), though 15% and 14% of total COVID-19 deaths occurred in the decedent’s home and a long-term care facility, respectively [7]. 

Advanced age and certain comorbid conditions are well-defined risk factors for developing severe COVID-19 disease and experiencing worse clinical outcomes [4, 8]. A large proportion of individuals ≥ 65 years have at least one comorbid condition and require long-term care, especially after a hospitalization. There are limited data related to the patient burden post-COVID-19 hospitalization in more recent years, including data related to loss of independence, mortality, readmission rates and healthcare resource utilization, especially in long-term care settings.

The objective of this study is to describe post-discharge care settings and patient mortality occurring after a COVID-19 hospitalization among adults aged ≥ 65 years in the US. A better understanding of the longitudinal COVID-19 patient journey will allow for more accurate estimations of mortality and burden, as well as potential guidance for public health policy.

## Methods

### Study design and data source

This is a retrospective observational study that utilized a 100% Medicare fee-for-service (FFS) dataset. This study uses secondary data and is exempt from Institutional Review Board review. The study time frame (March 2023–August 2024) was selected to provide the most recently available data. Eligible patients had an inpatient admission for COVID-19 from September 2023–February 2024. The index date was the date of hospital discharge. The baseline period was six months prior to hospital admission and patients were followed until the first occurrence of six months post-index, death, or end of enrollment. Refer to Figure S1 for the study design schema.

### Study population

Patients included in the analysis had to meet the following inclusion criteria to be eligible: age-based eligibility for Medicare (≥ 65 years old); inpatient admission with COVID-19 (ICD-10-CM code U07.1 in the first or second position) between September 1, 2023, and February 29, 2024; and ≥ 6 months of enrollment in Medicare parts A, B, and D prior to the index hospitalization.

Exclusion criteria included any of the following: admission to the hospital for non-COVID-19-related reasons (e.g., unintentional injury, physical trauma, or poisoning) coupled with a positive COVID-19 test result, prior hospital admission for COVID-19 during the pre-index baseline period (six months pre-hospitalization), enrollment in Medicare Part C (Medicare Advantage), or a planned readmission per discharge code on index admission claim.

### Patient demographics and clinical and hospitalization characteristics

Patient demographics captured included age, sex, race/ethnicity, census region, and Medicaid-Medicare dual enrollment status. Clinical characteristics were captured in the baseline period and comprised of the following: high-risk comorbid conditions (as defined by the Center for Disease Control and Prevention [CDC] [9]), Charlson comorbidity index (CCI) score [10, 11], pre-hospitalization care setting, receipt of any COVID-19 vaccination, COVID-19 infection, and receipt of antiviral treatment. Comorbid conditions were identified in all FFS files and vaccination data were identified using Current Procedural Terminology-4 (CPT-4) and Healthcare Common Procedure Coding System (HCPCS) codes. The pre-hospitalization care setting was defined as the location of the most recent claim within the 30 days prior to the index hospital admission. If no claims were found, the patient was assumed to be at home under self-care. If multiple claims were found on the date closest to index hospital admission, the following hierarchy was applied in accordance with level of care: hospice, inpatient admission, skilled nursing facility (SNF)/other facilities, home (under care), and home (self-care).

Characteristics of the index hospitalization included COVID-19 hospitalization severity, which was evaluated based on intensive care unit (ICU) admission and invasive mechanical ventilation (IMV) usage and grouped into three mutually exclusive categories: general ward, ICU (without IMV), and IMV (with or without ICU). ICU admission and IMV usage were identified by revenue codes, HCPCS codes, and/or CPT-4 codes. Patients without codes for ICU or IMV were classified as general ward. Length of stay (LOS) for the index hospitalization and inpatient antiviral treatment were also assessed.

Patient demographics, clinical, and hospitalization characteristics were stratified by post-discharge care setting. Hospitalization characteristics were also stratified by COVID-19 hospitalization severity.

### Measured outcomes

Patient outcomes of interest included first post-discharge care setting, hospital readmissions (COVID-19-related and all-cause), and mortality. Hospital readmissions and mortality were stratified by post-discharge care setting, COVID-19 hospitalization severity, and age group. The follow-up time periods of interest for these outcomes were 30, 60, 90, and 180 days.

The first post-discharge care setting was identified using claims within a 7-day window following hospital discharge. The claim dated closest to discharge was used. If multiple claims were found on the same date post-discharge, the location of the claim with the latest end date was used. If multiple claims found on the same date post-discharge had the same start and through date, the following hierarchy was applied to identify the discharge location: hospice, inpatient readmission, SNF/other facilities, home (under-care), home (self-care). If no claims were present, a patient referral location available on inpatient claims was used to determine the post-discharge care setting. Post-discharge care settings were grouped into 3 categories: home (self-care), home (under care), and any healthcare facility.

“Home (self-care)” was assigned if there were no claims for another location within seven days post-discharge or if discharge status stated home with no care. “Home (under care)” included those with a home health claim within seven days post-discharge as well as those who did not have any claims across any setting within those seven days if their discharge status stated home with care. “Any healthcare facility” was assigned for those with a claim within seven days of discharge for any other location, including an SNF, inpatient rehabilitation facility (IRF), hospice, intermediate care facility (ICF), long-term care facility (LTCH), psychiatric inpatient unit, or other type of health care institutions as defined by discharge status code. The second post-discharge care setting was identified using claims within 30 days of the end date of the first post-discharge setting. If a claim indicating a subsequent discharge setting was observed within 30 days, that was assigned as the second post-discharge care setting. If no claims were observed within 30 days of the end date of the first post-discharge location and ≥ 30 days of follow-up remained, home (self-care) was assigned. Patients with < 30 days of follow-up after the first post-discharge setting and no claims indicating a subsequent discharge setting were assigned no second setting. In the absence of evidence of a subsequent transition, it can be assumed that these patients remained in the first post-discharge care setting until the end of follow-up or death.

All-cause and COVID-19-related (cause-specific) readmissions were identified as any subsequent hospitalization to a critical access hospital or inpatient hospital (psychiatric inpatient facilities and IRFs were not included), within 30, 60, 90, and 180 days of discharge. Cause-specific readmissions followed the same methodology, requiring COVID-19 codes to be in the first or second diagnosis position. As follow-up time is variable within the population, readmission rates are reported as a percentage of those who were readmitted within the periods of interest (30, 60, 90, and 180 days), conditional on having the appropriate amount of follow-up time for each.

Mortality (all-cause) was identified through the master beneficiary summary file in the FFS dataset using the date of death. As follow-up time is variable within the population, mortality was reported as a percentage of those who died within the periods of interest (30, 60, 90, and 180 days), conditional on having the appropriate amount of follow-up time for each.

### Statistical analysis

Descriptive statistics were utilized to summarize patient demographic, clinical, and hospitalization characteristics, post-discharge care setting, mortality, and readmissions. Counts and percentages were reported for categorical variables. Continuous variables were summarized using means and standard deviations (SD) as well as medians and interquartile ranges (IQR). No formal statistical testing or between-group comparisons were performed.

## Results

### Study population

A total of 67,358 patients met the inclusion criteria and were included in the analysis (see Table S1 for details of patient attrition). Overall, most patients were female (55.6%) and white (84.9%) with an average age of 80.8 (SD: 8.1) years. The mean CCI score was 3.7 (SD: 2.7) and nearly all patients had at least one high-risk condition, as defined by the CDC [9], that increases likelihood of severe COVID-19 (excluding age; 96.4%). See Table 1 for patient demographics and Table S2 for disease characteristics.

**Table 1:** Patient demographic, clinical, and hospitalization characteristics stratified by post-discharge care location

		First Post-Discharge Care Setting		
Characteristic	Overall	Home (self-care)	Home (under care)	Any healthcare facility^1^
Patient count N (%)	67,358 (100.0%)	25,962 (38.5%)	17,248 (25.6%)	24,148 (35.9%)
*Demographic Characteristics*				
Age^2^				
Mean (SD)	80.8 (8.1)	78.2 (7.5)	82.0 (7.8)	82.6 (8.1)
Age group^2^				
65–75 years	16,666 (24.7%)	9089 (35.0%)	3236 (18.8%)	4341 (18.0%)
75+ years	50,692 (75.3%)	16,873 (65.0%)	14,012 (81.2%)	19,807 (82.0%)
Beneficiary sex				
Female	37,427 (55.6%)	13,728 (52.9%)	9952 (57.7%)	13,747 (56.9%)
Male	29,931 (44.4%)	12,234 (47.1%)	7296 (42.3%)	10,401 (43.1%)
Race/ethnicity				
White, non-Hispanic	57,169 (84.9%)	22,069 (85.0%)	14,427 (83.6%)	20,673 (85.6%)
Black, non-Hispanic	3821 (5.7%)	1260 (4.9%)	1038 (6.0%)	1523 (6.3%)
Hispanic all races	2942 (4.4%)	1128 (4.3%)	849 (4.9%)	965 (4.0%)
Asian/Pacific Islander, non-Hispanic	1780 (2.6%)	662 (2.5%)	555 (3.2%)	563 (2.3%)
American Indian, non-Hispanic	308 (0.5%)	193 (0.7%)	49 (0.3%)	66 (0.3%)
Other/Unknown	1338 (2.0%)	650 (2.5%)	330 (1.9%)	358 (1.5%)
US census region				
Northeast	18,311 (27.2%)	6078 (23.4%)	5388 (31.2%)	6845 (28.3%)
Midwest	16,509 (24.5%)	7162 (27.6%)	3526 (20.4%)	5821 (24.1%)
South	21,888 (32.5%)	8328 (32.1%)	5593 (32.4%)	7967 (33.0%)
West	10,650 (15.8%)	4394 (16.9%)	2741 (15.9%)	3515 (14.6%)
Medicaid-Medicare dual enrollment at index				
Full dual	12,105 (18.0%)	3089 (11.9%)	2813 (16.3%)	6203 (25.7%)
Nondual	53,123 (78.9%)	22,070 (85.0%)	13,828 (80.2%)	17,225 (71.3%)
Partial dual	2130 (3.2%)	803 (3.1%)	607 (3.5%)	720 (3.0%)
*Clinical Characteristics*				
High-risk comorbidities				
Any high-risk condition^3^	64,919 (96.4%)	24,697 (95.1%)	16,768 (97.2%)	23,454 (97.1%)
CCI score at baseline				
Mean (SD)	3.7 (2.7)	3.2 (2.5)	3.9 (2.7)	4.1 (2.7)
Pre-hospitalization care setting				
Any healthcare facility	9967 (14.8%)	1621 (6.2%)	1366 (7.9%)	6980 (28.9%)
Home (under care)	9125 (13.5%)	1264 (4.9%)	4092 (23.7%)	3769 (15.6%)
Home (self-care)	48,266 (71.7%)	23,077 (88.9%)	11,790 (68.4%)	13,399 (55.5%)
Vaccination in baseline period				
COVID-19 vaccination during baseline	10,306 (15.3%)	4329 (16.7%)	2606 (15.1%)	3371 (14.0%)
No COVID-19 vaccination during baseline	57,052 (84.7%)	21,633 (83.3%)	14,642 (84.9%)	20,777 (86.0%)
Preadmission COVID-19 infection				
COVID-19 in 180 days prior to admission	31,325 (46.5%)	11,692 (45.0%)	7953 (46.1%)	11,680 (48.4%)
No baseline COVID-19	36,033 (53.5%)	14,270 (55.0%)	9295 (53.9%)	12,468 (51.6%)
Preadmission antiviral treatment				
Baseline AV treatment	9308 (13.8%)	3966 (15.3%)	2452 (14.2%)	2890 (12.0%)
No baseline AV treatment	58,050 (86.2%)	21,996 (84.7%)	14,796 (85.8%)	21,258 (88.0%)
*Hospitalization Characteristics*				
COVID-19 hospitalization severity				
General ward	60,972 (90.5%)	24,181 (93.1%)	15,689 (91.0%)	21,102 (87.4%)
ICU without IMV	2965 (4.4%)	934 (3.6%)	718 (4.2%)	1313 (5.4%)
IMV with or without ICU	3421 (5.1%)	847 (3.3%)	841 (4.9%)	1733 (7.2%)
Length of stay (days)				
Mean (SD)	5.8 (4.9)	4.1 (2.5)	5.6 (3.6)	7.7 (6.7)
Median (IQR)	5.0 (3.0–7.0)	4.0 (3.0–5.0)	5.0 (3.0–7.0)	6.0 (4.0–9.0)
Inpatient antiviral treatment				
Antiviral treatment during index admission	33,046 (49.1%)	12,101 (46.6%)	9174 (53.2%)	11,771 (48.7%)
No Antiviral treatment during index admission	34,312 (50.9%)	13,861 (53.4%)	8074 (46.8%)	12,377 (51.3%)

Additional comorbidity data is presented in Supplemental Table S2

*AV* Antiviral, *CCI* Charlson Comorbidity Index, *ICU* Intensive care unit, *IMV* Invasive mechanical ventilation, *IQR* Interquartile range, *SD* Standard deviation, *US* United States

^1^Any healthcare facility included: skilled nursing facilities, inpatient rehabilitation facilities, hospice, intermediate care facilities, long-term care hospitals, psychiatric inpatient unit, and other types of health care institutions

^2^Age as of index date (date of hospital discharge)

The pre-hospitalization care setting for the majority of patients (71.7%) was home (self-care); 13.5% of patients were home under care and 14.8% were admitted from a healthcare facility. During the baseline period, 15.3% of patients received a COVID-19 vaccine. During the index hospitalization, 90.5% of patients were admitted to the general ward, 4.4% to the ICU without IMV, and 5.1% received IMV (with or without ICU). The average LOS was 5.8 (SD: 4.9) days (median 5.0, IQR: 3.0–7.0 days), with patients receiving IMV (with or without ICU) experiencing the longest LOS (average 9.3, SD: 8.0 days) (Table 2).

**Table 2:** Hospitalization characteristics stratified by COVID-19 hospitalization severity

Characteristic		COVID-19 Hospitalization Severity		
	Overall	General Ward	ICU without IMV	IMV with or without ICU
Patient Count N (%)	67,358 (100.0%)	60,972 (90.5%)	2965 (4.4%)	3421 (5.1%)
Length of stay, days (continuous)				
Mean (SD)	5.8 (4.9)	5.5 (4.6)	7.7 (5.3)	9.3 (8.0)
Median (IQR)	5.0 (3.0–7.0)	4.0 (3.0–6.0)	6.0 (4.0–10.0)	7.0 (5.0–11.0)
Length of stay, days (categorical)				
1 to 3 days	19,305 (28.7%)	18,464 (30.3%)	407 (13.7%)	434 (12.7%)
4 to 6 days	30,158 (44.8%)	27,853 (45.7%)	1158 (39.1%)	1147 (33.5%)
7+ days	17,895 (26.6%)	14,655 (24.0%)	1400 (47.2%)	1840 (53.8%)
Inpatient antiviral treatment				
Antiviral treatment during index admission	33,046 (49.1%)	29,309 (48.1%)	1511 (51.0%)	2226 (65.1%)
No Antiviral treatment during index admission	34,312 (50.9%)	31,663 (51.9%)	1454 (49.0%)	1195 (34.9%)

*ICU* Intensive care unit, *IMV* Invasive mechanical ventilation, *IQR* Interquartile range, *SD* Standard deviation

### Post-discharge care setting

**Fig. 1:**
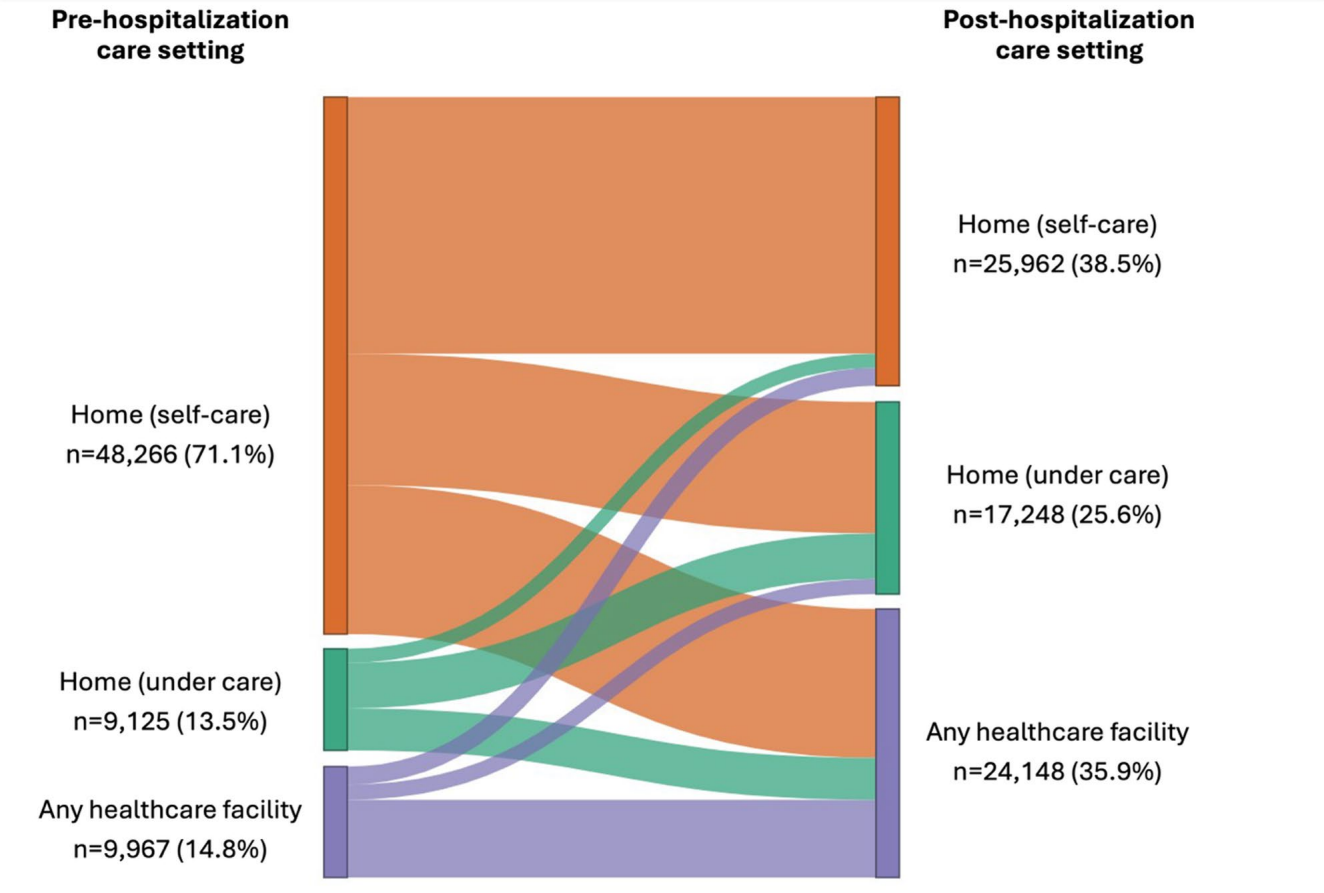
Transitions of care settings pre-hospitalization to post-hospitalization

Patients were discharged to home (self-care) (38.5%), home (under care) (25.6%), or any healthcare facility (35.9%). Transitions from the pre- to post-hospitalization setting can be seen in Fig. 1 and Table S3. The percentage of patients who resided in their home under care increased from pre- to post-hospitalization (13.5% to 25.6%), as did the percentage of patients in a healthcare facility (14.8% to 35.9%). The percentage of patients who resided in their home (self-care) decreased from 71.7% to 38.5%. Less than half (47.8%) of patients who were home under self-care pre-hospitalization returned there upon discharge. Of those eligible for an increased level of care (i.e., those who resided at home [self-care or under care]), 50.5% required increased care following hospitalization for COVID-19. Second post-discharge care settings were home (self-care) for 23.7% of patients, home (under care) for 14.1%, and any healthcare facility for 32.1%, while 30.1% had no second post-discharge care setting identified (i.e., remained in the same setting until end of follow up) (Table S4). After taking into account those who remained in the same setting, 47.0% of patients’ second post-discharge care setting was home (self-care), 15.6% was home (under care), and 37.4% was any healthcare facility.

The average age was older for patients discharged to any healthcare facility (82.6 [SD: 8.1] years) than those discharged to home (self-care) (78.2 [SD: 7.5] years) or home (under care) (82.0 [SD: 7.8] years). Patients discharged to any healthcare facility also had a higher baseline CCI average (4.1 [SD: 2.7]) compared to those discharged to home under care (3.2 [SD: 2.5]) and home (self-care) (3.9 [SD: 2.7]). The subgroup discharged to any healthcare facility also had a higher proportion of patients requiring IMV (with or without ICU) (7.2% vs. 3.3% and 4.9%, respectively) and a longer average LOS during the index hospitalization (7.7 [SD: 6.7] days vs. 4.1 [SD: 2.5] and 5.6 [SD: 3.6], respectively).

### COVID-19-related hospital readmissions

A total of 2,543 (4.5%) of patients were readmitted to the hospital for COVID-19 within 6 months of discharge; 3.2% of readmissions occurred within 30 days, 3.7% within 60 days and 4.0% within 90 days. Rates of COVID-related readmissions within 6 months were higher for those discharged to any healthcare facility (6.0%) compared to home (self-care) (3.7%) and home (under-care) (4.4%). This trend was observed at all time points (Table 3).

**Table 3:** COVID-19-related hospital readmissions at various time points stratified by post-discharge care setting

		First Post-Discharge Care Setting		
	Overall	Home (self-care)	Home (under care)	Any healthcare facility^1^
Patient count N (%)	67,358 (100.0%)	25,962 (38.5%)	17,248 (25.6%)	24,148 (35.9%)
**Patients readmitted within 30 days or had at least 30 days follow-up**				
Was not readmitted within 30 days follow-up	61,248 (96.8%)	24,910 (97.3%)	16,276 (97.0%)	20,062 (96.1%)
Readmitted between discharge and 30 days follow-up	2025 (3.2%)	699 (2.7%)	512 (3.0%)	814 (3.9%)
**Patients readmitted within 60 days or had at least 60 days follow-up**				
Was not readmitted within 60 days follow-up	58,832 (96.3%)	24,476 (96.9%)	15,737 (96.5%)	18,619 (95.4%)
Readmitted between discharge and 60 days follow-up	2262 (3.7%)	785 (3.1%)	574 (3.5%)	903 (4.6%)
**Patients readmitted within 90 days or had at least 90 days follow-up**				
Was not readmitted within 90 days follow-up	57,169 (96.0%)	24,136 (96.7%)	15,390 (96.2%)	17,643 (94.9%)
Readmitted between discharge and 90 days follow-up	2383 (4.0%)	828 (3.3%)	608 (3.8%)	947 (5.1%)
**Patients readmitted within 180 days or had at least 180 days follow-up**				
Was not readmitted within 180 days follow-up	53,434 (95.5%)	23,301 (96.3%)	14,435 (95.6%)	15,698 (94.0%)
Readmitted between discharge and 180 days follow-up	2543 (4.5%)	888 (3.7%)	659 (4.4%)	996 (6.0%)

Readmissions are reported as a percentage of those who had the full amount of follow up time at each period of interest (30, 60, 90, and 180 days) and those who were readmitted within said period of interest. Rates at each time period are cumulative

^1^Any healthcare facility included: skilled nursing facilities, inpatient rehabilitation facilities, hospice, intermediate care facilities, long-term care hospitals, psychiatric inpatient unit, and other types of health care institutions

Readmission rates for COVID-19 within six months post-discharge were also higher for patients who received IMV during the index hospitalization compared to those admitted to the general ward (4.4%) or the ICU (5.2%) on index hospitalization (Table 4). Among patients aged 65–74 years, 4.4% were readmitted for COVID-19 within 6 months post-discharge; a similar rate (4.6%) of those aged ≥ 75 years were readmitted. All-cause hospital readmissions are reported in Table S4 and Table S5.

**Table 4:** COVID-19-related hospital readmissions at various time points stratified by COVID-19 hospitalization severity and age group

		COVID-19 Hospitalization Severity			Age Group	
	Overall	General Ward	ICU without IMV	IMV with or without ICU	Age 65–74	Age 75+
Patient count N (%)	67,358 (100.0%)	60,972 (90.5%)	2965 (4.4%)	3421 (5.1%)	16,666 (24.7%)	50,692 (75.3%)
**Patients readmitted within 30 days or had at least 30 days follow-up**						
Was not readmitted within 30 days follow-up	61,248 (96.8%)	55,878 (96.9%)	2553 (96.4%)	2817 (96.0%)	15,509 (96.7%)	45,739 (96.8%)
Readmitted between discharge and 30 days follow-up	2025 (3.2%)	1814 (3.1%)	95 (3.6%)	116 (4.0%)	532 (3.3%)	1493 (3.2%)
**Patients readmitted within 60 days or had at least 60 days follow-up**						
Was not readmitted within 60 days follow-up	58,832 (96.3%)	53,813 (96.4%)	2405 (95.9%)	2614 (95.0%)	15,054 (96.2%)	43,778 (96.3%)
Readmitted between discharge and 60 days follow-up	2262 (3.7%)	2020 (3.6%)	103 (4.1%)	139 (5.0%)	598 (3.8%)	1664 (3.7%)
**Patients readmitted within 90 days or had at least 90 days follow-up**						
Was not readmitted within 90 days follow-up	57,169 (96.0%)	52,346 (96.1%)	2320 (95.6%)	2503 (94.3%)	14,712 (95.9%)	42,457 (96.0%)
Readmitted between discharge and 90 days follow-up	2383 (4.0%)	2124 (3.9%)	108 (4.4%)	151 (5.7%)	629 (4.1%)	1754 (4.0%)
**Patients readmitted within 180 days or had at least 180 days follow-up**						
Was not readmitted within 180 days follow-up	53,434 (95.5%)	49,034 (95.6%)	2153 (94.8%)	2247 (93.5%)	13,982 (95.6%)	39,452 (95.4%)
Readmitted between discharge and 180 days follow-up	2543 (4.5%)	2271 (4.4%)	117 (5.2%)	155 (6.5%)	650 (4.4%)	1893 (4.6%)

Readmissions are reported as a percentage of those who had the full amount of follow up time at each period of interest (30, 60, 90, and 180 days) and those were readmitted within said period of interest. Rates at each time period are cumulative

*ICU* Intensive care unit, *IMV* Invasive mechanical ventilation

### All-cause mortality

**Fig. 2:**
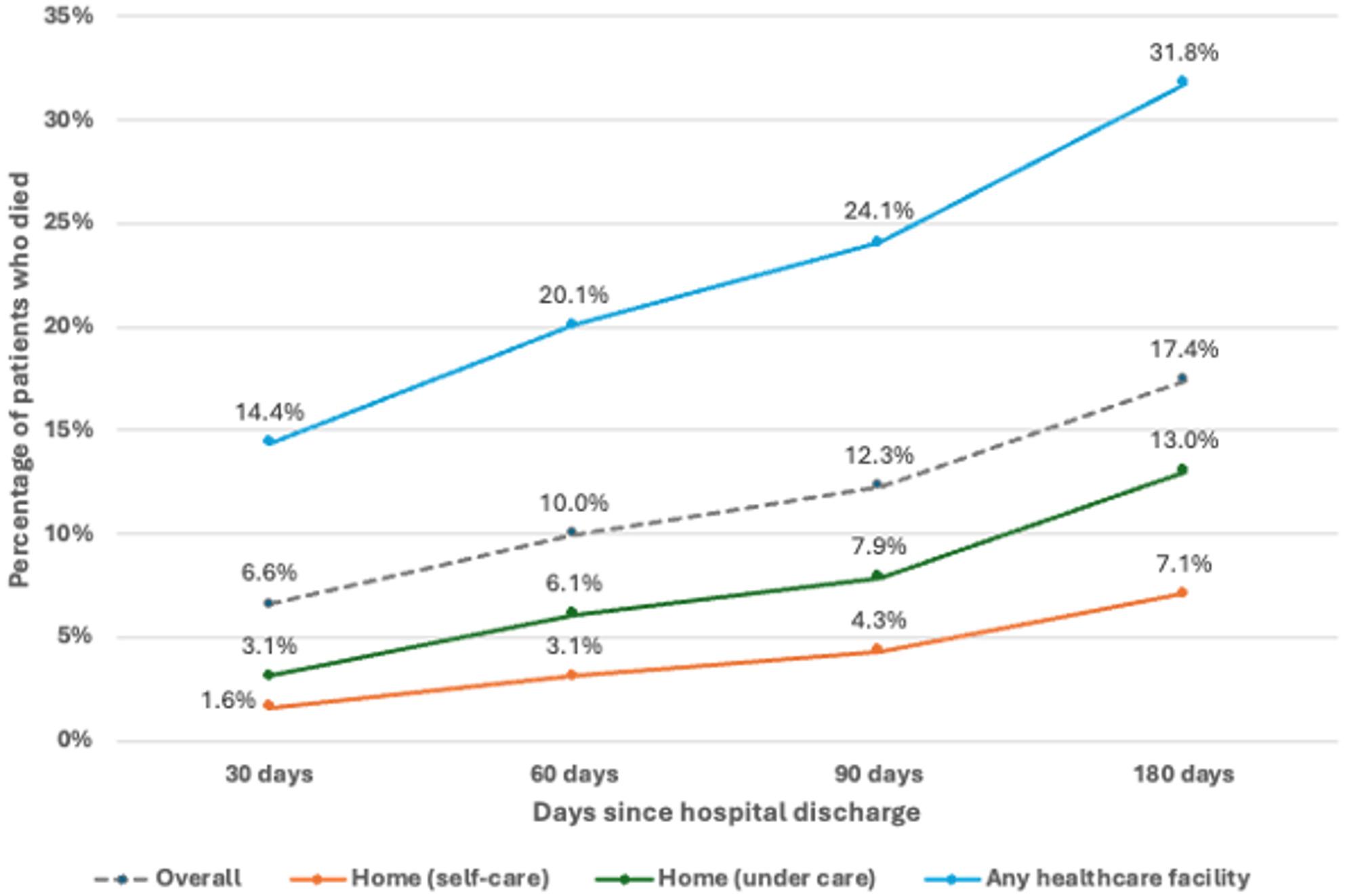
Mortality at various time points stratified by post-discharge care setting

A total of 11,658 (17.4%) patients died within six months of hospital discharge; 6.6% of the deaths occurred within 30 days, 10.0% within 60 days, and 12.3% within 90 days post-hospitalization (Fig. 2; Table S6). Mortality rates varied by post-discharge care setting (Fig. 2). A total of 7.1% of patients discharged to home (self-care) died within six months of hospital discharge, whereas 13.0% of patients discharged to home (under care) and 31.8% discharged to any healthcare facility died within six months (Table S6).

Of those admitted to the general ward on index hospitalization, 16.4% died within six months of discharge, compared to 23.9% of those admitted to the ICU and 31.0% who received IMV (Table 5). Mortality rates within six months post-discharge were higher in patients aged ≥ 75 years than those aged 64–74 years (19.1% and 12.4%, respectively). This trend was similar regardless of post-discharge care location (Table 5).

**Table 5:** Mortality at various time points stratified by COVID-19 hospitalization severity and age group

Characteristic		COVID-19 Hospitalization Severity			Age Group	
	Overall	General Ward	ICU without IMV	IMV with or without ICU	Age 65–74	Age 75+
Patient count N (%)	67,358 (100.0%)	60,972 (90.5%)	2965 (4.4%)	3421 (5.1%)	16,666 (24.7%)	50,692 (75.3%)
**Patients who died within 30 days or had at least 30 days follow-up**						
Did not die within 30 days follow-up	62,877 (93.4%)	57,350 (94.1%)	2627 (88.7%)	2900 (84.8%)	15,964 (95.9%)	46,913 (92.6%)
Died between discharge and 30 days follow-up	4426 (6.6%)	3572 (5.9%)	336 (11.3%)	518 (15.2%)	690 (4.1%)	3736 (7.4%)
**Patients who died within 60 days or had at least 60 days follow-up**						
Did not die within 60 days follow-up	60,533 (90.0%)	55,349 (90.9%)	2478 (83.8%)	2706 (79.2%)	15,532 (93.3%)	45,001 (88.9%)
Died between discharge and 60 days follow-up	6711 (10.0%)	5522 (9.1%)	480 (16.2%)	709 (20.8%)	1108 (6.7%)	5603 (11.1%)
**Patients who died within 90 days or had at least 90 days follow-up**						
Did not die within 90 days follow-up	58,893 (87.7%)	53,897 (88.6%)	2397 (81.1%)	2599 (76.2%)	15,197 (91.4%)	43,696 (86.4%)
Died between discharge and 90 days follow-up	8279 (12.3%)	6906 (11.4%)	559 (18.9%)	814 (23.8%)	1423 (8.6%)	6856 (13.6%)
**Patients who died within 180 days or had at least 180 days follow-up**						
Did not die within 180 days follow-up	55,154 (82.6%)	50,589 (83.6%)	2235 (76.1%)	2330 (69.0%)	14,463 (87.6%)	40,691 (80.9%)
Died between discharge and 180 days follow-up	11,658 (17.4%)	9912 (16.4%)	701 (23.9%)	1045 (31.0%)	2049 (12.4%)	9609 (19.1%)

Mortality is reported as a percentage of those who had the full amount of follow up time at each period of interest (30, 60, 90, and 180 days) and those that died within said period of interest. Rates at each time period are cumulative

*ICU* Intensive care unit, *IMV* Invasive mechanical ventilation

## Discussion

In this analysis of older patients with a high prevalence (96.4%) of high-risk comorbidities, COVID-19 hospitalization served as a catalyst for requiring increased levels of care and a loss of independence for some patients upon discharge, as exhibited by less than half of patients who resided in their home under self-care prior to being hospitalized with COVID-19 returning to their home without care post-discharge. When examining second post-discharge care locations, more patients were able to return home without care, although care requirements for many patients remained higher than pre-hospitalization levels. Increased care needs were most evident among patients with more severe COVID-19 during the index hospitalization and who were older (aged ≥ 75 years). Individuals in this study spent several days (5.8 on average) in the hospital despite most being admitted to the general ward and considered to have low-severity COVID-19. Increased readmission rates were observed for those who transitioned to a healthcare facility and those who were admitted to the ICU or required IMV during the index hospitalization. The mortality rate within six months of hospital discharge was 17.4%. This increased for those of advanced age, with more severe COVID-19, and for those who required increased care upon discharge.

It is worth noting that a nontrivial number (*n* = 8,601) of patients were admitted for reasons unrelated to COVID-19 and incidentally tested positive during the admission (Table S1). These individuals were excluded from the analysis. Incidental COVID-19 infections, particularly in the context of variants associated with milder disease, may not carry the same risk of functional decline as hospitalizations primarily attributable to COVID-19. Our findings reflect outcomes in patients whose hospitalization was directly attributable to COVID-19.

Data are limited regarding the patient journey following a COVID-19 hospitalization, especially for elderly patients in more recent years. Our findings align with a previous study by Yehoshua et al., which demonstrated that more severe COVID-19 (as measured by ICU admission and IMV usage) and advanced age were associated with worse outcomes (increased LOS, higher inpatient mortality and hospitalization cost) [12]. Our findings also align with a recently published study by Roberts et al., which reported that approximately 50% of high-risk Medicare patients hospitalized with COVID-19 were discharged home under self-care in early 2022 [13]. 

Readmission rates from our study align with findings from other research in older patients with COVID-19. For example, the Agency for Healthcare Research and Quality reported that the rate of 30-day all-cause readmissions for Medicare patients aged ≥ 65 years was 15.9% on average during the pre-pandemic stage (2016–2019) [14] and Oseran et al. reported a 30-day all-cause readmission for Medicare patients admitted to the hospital with COVID-19 (2020–2022) of 16.0% [15]. The 30-day all-cause readmission rate from our study was 17.6% (Table S4). Both all-cause and COVID-19-related readmissions increased with higher post-discharge care requirements and COVID-19 hospitalization severity. Readmissions increased slightly in the ≥ 75 years age group. Notably, COVID-19 readmissions represented approximately 10% of all-cause readmissions 180 days post-discharge.

Our study found that a total of 11,658 (17.4%) patients died within 180 days of being discharged from a COVID-19 hospitalization, with most deaths occurring within 30 days, suggesting that the immediate period post-hospital discharge represents a particularly vulnerable time for older patients. Mortality was highest in patients requiring increased levels of care post-discharge, those with higher COVID-19 severity (i.e., required ICU stay or IMV), and those aged ≥ 75 years.

Our findings align with other studies that have reported increased mortality in older patients after a COVID-19 hospitalization [13, 15, 16]. A study of US veterans hospitalized with COVID-19 in 2020 found a three-fold increased risk for death in the first year post-hospitalization compared to controls [16]. Oseran et al. examined all-cause mortality rates in Medicare patients post-COVID-19 hospitalization compared to historical control patients with influenza. The average 30-day all-cause mortality was 10.9% and 3.9%, respectively, and the 180-day mortality rate was 19.1% versus 10.5% - nearly double for the COVID-19 cohort compared to the influenza cohort [15]. Roberts et al. reported a 30-day all-cause mortality rate for high-risk Medicare patients hospitalized for COVID-19 in December 2022 of 6.25% [13]. This estimate aligns closely with the 30-day all-cause mortality rate reported in our study (6.6%).

Future research should examine mortality among beneficiaries without a COVID-19 hospitalization, as well as comparisons with other respiratory conditions or all-cause hospitalizations, to provide additional context for understanding the outcomes observed in this study. Additionally, studies with longer follow-up are needed to better characterize long-term care trajectories and to determine the duration of increased care needs. Furthermore, evaluating the role of preventive health measures, particularly antiviral use and vaccination, in shaping subsequent outcomes, including transitions to post-discharge care settings, is an important direction for future research.

### Limitations

This study has a number of limitations. First, the mortality data reported in this study are for all causes as the specific cause of death is not reported in the data source. Further investigation is warranted to determine mortality rates due to COVID-19 in the post-hospitalization setting. The CDC suggests that mortality due to COVID-19 is often underreported and only counting deaths in which COVID-19 was recorded on the death certificate would substantially understate the true impact [17]. As such, adjustments are made in burden estimates by the CDC to account for underreporting, with preliminary estimates in the 2024–2025 season of 29,000–47,000 COVID-19 deaths (data as of April 26, 2025) [1].

Second, it is possible that patients transitioned to higher levels of care for reasons unrelated to COVID-19. Despite this potential limitation, the comparison to pre-hospitalization locations in our analysis indicates that a COVID-19 hospitalization serves as a catalyst for requiring higher levels of care for many patients. Third, pre-hospital and post-discharge settings were identified using claims data. The care setting identified in claims closest to admission/discharge and discharge status was used if no claims within the period in question were available. If there were no claims within seven days and no care setting was identified on discharge status, the care setting was assumed to be home (self-care), but this assumption may not be accurate. Not every patient had a post-discharge care claim, and we acknowledge that discharge status may not always reflect the care actually received, particularly when no subsequent claim is available. However, the discharge disposition indicates the level of care intended by the treating provider at the time of discharge and therefore provides meaningful information about the planned post-acute care arrangement. Notably, the majority (71%) of the individuals classified as ‘home (under care)’ had a home health claim recorded. Fourth, all healthcare facilities were grouped into a single, broad category to help with interpretation of results, but we acknowledge that there are inherent differences in various healthcare settings. A more granular look at post-discharge care locations by type of care facility can be found in Table S7. Finally, vaccination data in the baseline period are likely underreported in this study due to the possibly that patients may have received a vaccine prior to the six-month baseline period as well as the potential to receive vaccination through sources not identified in claims [18]. 

## Conclusion

This study showed that hospitalization for COVID-19 in the ≥ 65-year-old population is associated with poor outcomes, including loss of independence and increased care requirements upon discharge, as well as increased mortality, and high rates of readmission to the hospital, even in recent seasons. The post-hospitalization period represents a particularly vulnerable time for elderly patients; particular attention during this transitional period is warranted to ensure that these patients receive required care. Lapo et al. reported that the loss of functionality experienced by elderly patients after a COVID-19 hospitalization can be improved with inpatient rehabilitation [19]. 

Beyond the loss of independence experienced by many older patients who transition to higher levels of care, whether transient or sustained, there are substantial cost implications. Further research is warranted to determine the economic impact of increased healthcare utilization required post-COVID-19 hospitalization. These findings underscore the continued importance of preventive measures aimed at reducing the risk of severe disease leading to hospitalization, such as vaccination and antiviral use. While our study did not evaluate these measures directly, they remain central components of broader public health strategies that may help mitigate downstream effects of infection.

## Supplementary Information

Supplementary Material 1.

## Data Availability

The data that support the findings of this study are available from The Centers for Medicare and Medicaid Services (CMS) and were obtained through a restricted data use agreement for the current study.

## Abbreviations

AV: Antiviral
CCI: Charlson Comorbidity Index
CDC: Centers for Disease Control and Prevention
COVID-19: Coronavirus disease 2019
CPT-4: Current Procedural Terminology, 4th edition
FFS: Fee-for-service
HCPCS: Healthcare Common Procedure Coding System
ICF: Intermediate care facility
ICU: Intensive care unit
IMV: Invasive mechanical ventilation
IQR: Interquartile range
IRF: Inpatient rehabilitation facility
LOS: Length of stay
LTCH: Long-term care facility
SNF: Skilled nursing facility
SD: Standard deviation
US: United States
