# Supplementary material for: Mortality and transitions-of-care after COVID-19 hospitalization among US Medicare patients: a retrospective claims analysis
Source: BMC Geriatr. 2026 May 28;26:985. doi: 10.1186/s12877-026-07700-7 (PMC13412174; doi:10.1186/s12877-026-07700-7)

**Supplemental Table S1:** Patient attrition

|  | **Remaining** | | **Excluded** |
| --- | --- | --- | --- |
| **Inclusion/Exclusion criteria** | **N** | **% of previous step** | **N** |
| Patients with Medicare Part A, B, and D as of September 2023 | 51,664,466 |  |  |
| Enrolled in Medicare for age as of September 1, 2023 | 45,263,075 | 87.61% | 6,401,391 |
| Located in 50 states and DC | 44,694,461 | 98.74% | 568,614 |
| Exclusion of patients with Medicare Part C (Medicare Advantage) at any point during study period (March 1, 2023 - August 31, 2024) | 16,918,222 | 37.85% | 27,776,239 |
| Had an inpatient admission with COVID-19 (U07.1) in the principal or second position between Sept 1, 2023, and Feb 29, 2024, with at least 1 overnight stay | 80,171 | 0.47% | 16,838,051 |
| Excluding patients who died on or before index (discharge date) | 77,673 | 96.88% | 2,498 |
| Continuous enrollment in Part A/B and D for 180 days before admission date | 76,879 | 98.98% | 794 |
| Patients admitted for non-COVID-19 reasons and tested positive for COVID-19 incidentally | 68,278 | 88.81% | 8,601 |
| Patients with COVID-19 hospitalization (any position) 180 days before admission date | 67,438 | 98.77% | 840 |
| Exclude patients with a planned readmission as their discharge status | 67,358 | 99.88% | 80 |

**Supplemental Table S2:** High-risk comorbidities by post-discharge care setting

|  | | **First Post-Discharge Care Setting** | | |
| --- | --- | --- | --- | --- |
| **Characteristic** | **Overall** | **Home  (self-care)** | **Home  (under care)** | **Any  healthcare  facility^1^** |
| Patient count  N (%) | **67358 (100.0%)** | **25962 (38.5%)** | **17248 (25.6%)** | **24148 (35.9%)** |
| **High-risk comorbidities** | | | | |
| **Any high-risk condition** | 64919 (96.4%) | 24697 (95.1%) | 16768 (97.2%) | 23454 (97.1%) |
| Asthma | 1700 (2.5%) | 806 (3.1%) | 468 (2.7%) | 426 (1.8%) |
| Cancer | 25728 (38.2%) | 10218 (39.4%) | 7037 (40.8%) | 8473 (35.1%) |
| Cerebrovascular disease | 31045 (46.1%) | 9182 (35.4%) | 8287 (48.0%) | 13576 (56.2%) |
| Chronic kidney disease | 25840 (38.4%) | 8711 (33.6%) | 7159 (41.5%) | 9970 (41.3%) |
| Chronic liver disease | 3331 (4.9%) | 1347 (5.2%) | 876 (5.1%) | 1108 (4.6%) |
| Chronic lung disease | 28912 (42.9%) | 10393 (40.0%) | 7865 (45.6%) | 10654 (44.1%) |
| Cystic fibrosis | 20 (0.0%) | 11 (0.0%) | n<11* | n<11* |
| Diabetes mellitus type I | 1642 (2.4%) | 552 (2.1%) | 464 (2.7%) | 626 (2.6%) |
| Diabetes mellitus type II | 27653 (41.1%) | 9991 (38.5%) | 7374 (42.8%) | 10288 (42.6%) |
| Disabilities | 25358 (37.6%) | 6252 (24.1%) | 6463 (37.5%) | 12643 (52.4%) |
| Heart conditions | 49979 (74.2%) | 18130 (69.8%) | 13282 (77.0%) | 18567 (76.9%) |
| HIV | 144 (0.2%) | 56 (0.2%) | 36 (0.2%) | 52 (0.2%) |
| Mental health conditions | 22405 (33.3%) | 6126 (23.6%) | 5676 (32.9%) | 10603 (43.9%) |
| Neurologic conditions limited to dementia | 8163 (12.1%) | 1159 (4.5%) | 1971 (11.4%) | 5033 (20.8%) |
| Overweight BMI^ | 7112 (10.6%) | 2888 (11.1%) | 1870 (10.8%) | 2354 (9.7%) |
| Obesity | 15826 (23.5%) | 6227 (24.0%) | 4172 (24.2%) | 5427 (22.5%) |
| Primary immunodeficiencies | 3545 (5.3%) | 1606 (6.2%) | 899 (5.2%) | 1040 (4.3%) |
| Sickle cell disease | 62 (0.1%) | 31 (0.1%) | 14 (0.1%) | 17 (0.1%) |
| Smoking current and former | 22478 (33.4%) | 8424 (32.4%) | 6059 (35.1%) | 7995 (33.1%) |
| Solid organ or blood stem cell  transplantation | 1236 (1.8%) | 686 (2.6%) | 295 (1.7%) | 255 (1.1%) |
| Substance use disorder | 2322 (3.4%) | 788 (3.0%) | 639 (3.7%) | 895 (3.7%) |
| Tuberculosis | 289 (0.4%) | 67 (0.3%) | 80 (0.5%) | 142 (0.6%) |
| Use of corticosteroids or other  immunosuppressive medications | 8505 (12.6%) | 3098 (11.9%) | 2527 (14.7%) | 2880 (11.9%) |

*The CMS cell size suppression policy stipulates that no cell (e.g. admissions, discharges, patients, services, etc.) containing a value of 1 to 10 can be reported directly. These counts have been censored as “n<11”

^Defined as BMI ≥ 25

BMI: body mass index, HIV: human immunodeficiency virus

1. Any healthcare facility included: skilled nursing facilities, inpatient rehabilitation facilities, hospice, intermediate care facilities, long-term care hospitals, psychiatric inpatient unit, and other types of health care institutions.

**Supplemental Table S3:** Pre- to post-hospitalization care setting transitions

|  |  | Pre-hospitalization care setting | | |  |
| --- | --- | --- | --- | --- | --- |
|  |  | Home (self-care) | Home (undercare) | Any healthcare facility | **Total** |
|  | Patient Count  N (%) | **N=48266 (71.7%)** | **N=9125 (13.5%)** | **N=9967 (14.8%)** | **(N=67358; 100%)** |
| Post-hospitalization care setting | Home (self-care) | 23077 (47.8%) | 1264 (13.9%) | 1621 (16.3%) | **25962 (38.5%)** |
|  | Home (under care) | 11790 (24.4%) | 4092 (44.8%) | 1366 (13.7%) | **17248 (25.6%)** |
|  | Any healthcare facility | 13399 (27.8%) | 3769 (41.3%) | 6980 (70.0%) | **24148 (35.9%)** |

**Supplemental Table S4: Second Post-Discharge Settings**

|  | **First post-discharge care setting** | | | |
| --- | --- | --- | --- | --- |
|  | **Overall** | **Home**  **(self-care)** | **Home**  **(under care)** | **Any healthcare facility** |
| **Patient Count** | **67358 (100.0%)** | **25962 (38.5%)** | **17248 (25.6%)** | **24148 (35.9%)** |
| **Second Post-Discharge Setting** | | | | |
| Home (self-care) | 15988 (23.7%) | 0 (0%) | 10423 (60.4%) | 5565 (23.0%) |
| Home (under care) | 9517 (14.1%) | 1897 (7.3%) | 0 (0%) | 7620 (31.6%) |
| Any healthcare facility | 21598 (32.1%) | 8393 (32.3%) | 5852 (33.9%) | 7353 (30.4%)* |
| No second post-discharge setting | 20255 (30.1%) | 15672 (60.4%) | 973 (5.6%) | 3610 (14.9%) |

*indicates patients who transferred from one type of healthcare facility to a different healthcare facility type

**Supplemental Table S5:** All-cause hospital readmissions by post-discharge care setting

|  |  | **First Post-Discharge Care Setting** | | |
| --- | --- | --- | --- | --- |
|  | **Overall** | **Home  (self-care)** | **Home  (under care)** | **Any healthcare facility^1^** |
| **Patient Count**  **N (%)** | **67358 (100.0%)** | **25962 (38.5%)** | **17248 (25.6%)** | **24148 (35.9%)** |
| **Patients readmitted within 30 days or had at least 30 days follow-up** | | | | |
| Was not readmitted within 30 days follow-up | 53195 (82.4%) | 22339 (86.5%) | 13946 (81.8%) | 16910 (78.0%) |
| Readmitted between discharge and 30 days follow-up | 11336 (17.6%) | 3482 (13.5%) | 3097 (18.2%) | 4757 (22.0%) |
| **Patients readmitted within 60 days or had at least 60 days follow-up** | | | | |
| Was not readmitted within 60 days follow-up | 47555 (74.6%) | 20736 (80.6%) | 12491 (73.9%) | 14328 (67.9%) |
| Readmitted between discharge and 60 days follow-up | 16184 (25.4%) | 4990 (19.4%) | 4420 (26.1%) | 6774 (32.1%) |
| **Patients readmitted within 90 days or had at least 90 days follow-up** | | | | |
| Was not readmitted within 90 days follow-up | 43571 (68.9%) | 19509 (76.0%) | 11424 (67.9%) | 12638 (61.0%) |
| Readmitted between discharge and 90 days follow-up | 19655 (31.1%) | 6152 (24.0%) | 5409 (32.1%) | 8094 (39.0%) |
| **Patients readmitted within 180 days or had at least 180 days follow-up** | | | | |
| Was not readmitted within 180 days follow-up | 35732 (57.5%) | 16846 (66.1%) | 9241 (55.6%) | 9645 (48.1%) |
| Readmitted between discharge and 180 days follow-up | 26401 (42.5%) | 8624 (33.9%) | 7379 (44.4%) | 10398 (51.9%) |

Note: Readmissions are reported as a percentage of those who had the full amount of follow up time at each period of interest (30, 60, 90, and 180 days) and those were readmitted within said period of interest. Rates at each time period are cumulative.

1. Any healthcare facility included: skilled nursing facilities, inpatient rehabilitation facilities, hospice, intermediate care facilities, long-term care hospitals, psychiatric inpatient unit, and other types of health care institutions.

**Supplemental Table S5:** All-cause hospital readmissions by COVID-19 hospitalization severity and age group

|  |  | **COVID-19 Hospitalization Severity** | | | | **Age Group** | |
| --- | --- | --- | --- | --- | --- | --- | --- |
|  | **Overall** | | **General Ward** | **ICU without IMV** | **IMV with or without ICU** | **Age 65-74** | **Age 75+** |
| **Patient Count**  N (%) | **67358 (100.0%)** | | **60972 (90.5%)** | **2965 (4.4%)** | **3421 (5.1%)** | **16666 (24.7%)** | **50692 (75.3%)** |
| **Patients readmitted within 30 days or had at least 30 days follow-up** | | | | | | | |
| Was not readmitted within 30 days follow-up | 53195(82.4%) | | 48838 (83.2%) | 2108 (77.3%) | 2249 (73.2%) | 13339 (81.8%) | 39856 (82.6%) |
| Readmitted between discharge and 30 days follow-up | 11336 (17.6%) | | 9894 (16.8%) | 618 (22.7%) | 824 (26.8%) | 2963 (18.2%) | 8373 (17.4%) |
| **Patients readmitted within 60 days or had at least 60 days follow-up** | | | | | | | |
| Was not readmitted within 60 days follow-up | 47555 (74.6%) | | 43859 (75.6%) | 1867 (69.7%) | 1829 (60.6%) | 12009 (74.1%) | 35546 (74.8%) |
| Readmitted between discharge and 60 days follow-up | 16184 (25.4%) | | 14187 (24.4%) | 810 (30.3%) | 1187 (39.4%) | 4191 (25.9%) | 11993 (25.2%) |
| **Patients readmitted within 90 days or had at least 90 days follow-up** | | | | | | | |
| Was not readmitted within 90 days follow-up | 43571 (68.9%) | | 40253 (69.9%) | 1702 (64.3%) | 1616 (54.0%) | 11040 (68.5%) | 32531 (69.1%) |
| Readmitted between discharge and 90 days follow-up | 19655 (31.1%) | | 17333 (30.1%) | 944 (35.7%) | 1378 (46.0%) | 5088 (31.5%) | 14567 (30.9%) |
| **Patients readmitted within 180 days or had at least 180 days follow-up** | | | | | | | |
| Was not readmitted within 180 days follow-up | 35732 (57.5%) | | 33099 (58.5%) | 1403 (54.1%) | 1230 (42.0%) | 9193 (57.7%) | 26539 (57.4%) |
| Readmitted between discharge and 180 days follow-up | 26401 (42.5%) | | 23512 (41.5%) | 1192 (45.9%) | 1697 (58.0%) | 6738 (42.3%) | 19663 (42.6%) |

ICU: intensive care unit, IMV: invasive mechanical ventilation

Note: Readmissions are reported as a percentage of those who had the full amount of follow up time at each period of interest (30, 60, 90, and 180 days) and those were readmitted within said period of interest. Rates at each time period are cumulative.

**Table S6:** Mortality at various time periods stratified by post-discharge care setting

|  |  | **First Post-Discharge Care Setting** | | |
| --- | --- | --- | --- | --- |
|  | **Overall** | **Home  (self-care)** | **Home  (under care)** | **Any healthcare facility^1^** |
| Patient count  N (%) | **67358 (100.0%)** | **25962 (38.5%)** | **17248 (25.6%)** | **24148 (35.9%)** |
| **Patients who died within 30 days or had at least 30 days follow-up** | | | | |
| Did not die within 30 days follow-up | 62877 (93.4%) | 25519 (98.4%) | 16711 (96.9%) | 20647 (85.6%) |
| Died between discharge and 30 days follow-up | 4426 (6.6%) | 425 (1.6%) | 526 (3.1%) | 3475 (14.4%) |
| **Patients who died within 60 days or had at least 60 days follow-up** | | | | |
| Did not die within 60 days follow-up | 60533 (90.0%) | 25114 (96.9%) | 16174 (93.9%) | 19245 (79.9%) |
| Died between discharge and 60 days follow-up | 6711 (10.0%) | 810 (3.1%) | 1050 (6.1%) | 4851 (20.1%) |
| **Patients who died within 90 days or had at least 90 days follow-up** | | | | |
| Did not die within 90 days follow-up | 58893 (87.7%) | 24792 (95.7%) | 15842 (92.1%) | 18259 (75.9%) |
| Died between discharge and 90 days follow-up | 8279 (12.3%) | 1114 (4.3%) | 1365 (7.9%) | 5800 (24.1%) |
| **Patients who died within 180 days or had at least 180 days follow-up** | | | | |
| Did not die within 180 days follow-up | 55154 (82.6%) | 23974 (92.9%) | 14887 (87.0%) | 16293 (68.2%) |
| Died between discharge and 180 days follow-up | 11658 (17.4%) | 1838 (7.1%) | 2227 (13.0%) | 7593 (31.8%) |

Note: Mortality is reported as a percentage of those who had the full amount of follow up time at each period of interest (30, 60, 90, and 180 days) and those that died within said period of interest. Rates at each time period are cumulative.

1. Any healthcare facility included: skilled nursing facilities, inpatient rehabilitation facilities, hospice, intermediate care facilities, long-term care hospitals, psychiatric inpatient unit, and other types of health care institutions.

**Supplemental Table S7:** First post-discharge care settings by facility type

| **Setting** | **Patient Count**  N (%) |
| --- | --- |
| **Home (self-care)** | **25962 (38.5%)** |
| Home (self-care) | 25692 (38.1%) |
| Left Against Medical Advice/Other | 270 (0.4%) |
| **Home (under care)** | **17248 (25.6%)** |
| **Any healthcare facility** | **24148 (35.9%)** |
| Skilled Nursing Facility (SNF) | 17252 (25.6%) |
| Inpatient Rehab Facility (IRF) | 2478 (3.7%) |
| Hospice | 2437 (3.6%) |
| Intermediate Care Facility (ICF) | 858 (1.3%) |
| Long-term Care Hospital (LTCH) | 426 (0.6%) |
| Inpatient psychiatric facility | 161 (0.2%) |
| Other type of health care institution^1^ | 536 (0.8%) |

1. Other type of health care institution includes the following discharge codes: transferred within or outside hospital, critical access hospital, and other type of health care institution

**Supplemental Figure S1:** Study design diagram


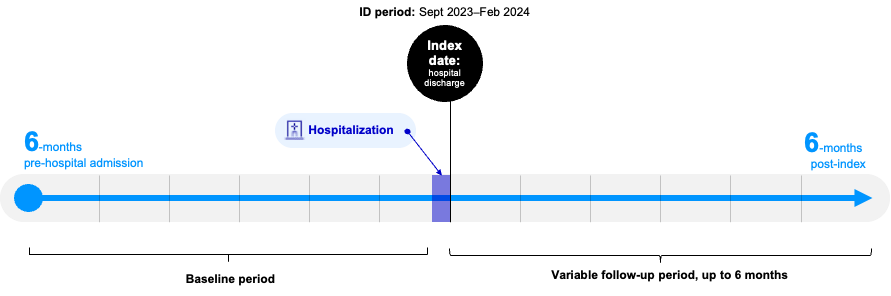

Supplement: Supplementary file 1 — Supplementary Material 1. [file 12877_2026_7700_MOESM1_ESM.docx]
